# Disease severity modifies the ceftazidime-avibactam exposure-response relationship in critically ill patients: a retrospective study

**DOI:** 10.3389/fphar.2026.1833093

**Published:** 2026-05-29

**Authors:** Shiyu Qian, Defang Xu, Yinqiu Xu, Hui Qi, Pei Liang

**Affiliations:** 1 Department of Pharmacy, Nanjing Drum Tower Hospital, Affiliated Hospital of Medical School, Nanjing University, Nanjing, China; 2 Department of Pharmacy, Nanjing Drum Tower Hospital, China Pharmaceutical University, Nanjing, China; 3 Department of Critical Care Medicine, Nanjing Drum Tower Hospital, Affiliated Hospital of Medical School, Nanjing University, Nanjing, China

**Keywords:** anti-bacterial agents, ceftazidime-avibactam (CAZ-AVI), intensive care units (ICU), sequential organ failure assessment scores (SOFA), therapeutic drug monitoring (TDM)

## Abstract

**Background:**

Ceftazidime-avibactam (CAZ-AVI) is a key agent for MDR-GNB, but significant pharmacokinetic (PK) variability in critically ill patients poses challenges to achieving optimal dosing.

**Objectives:**

To investigate the association and influencing factors between CAZ-AVI serum concentration and clinical efficacy in critically ill patients, and specifically to evaluate the influence of disease severity on this relationship, with the aim of identifying a therapeutic target for critically ill patients with different disease severity.

**Methods:**

A retrospective study was conducted of 168 critically ill patients receiving ceftazidime/avibactam between 2021 and 2025. The relationship between plasma drug concentration, clinical efficacy and disease severity was evaluated.

**Results:**

168 patients were included. C_min_ of CAZ and AVI was significantly higher in the effective group than in the ineffective group (CAZ: 39.68 mg/L vs. 26.11 mg/L, P < 0.001; AVI: 8.29 mg/L vs. 4.92 mg/L, P = 0.003). Multivariate analysis identified that CAZ C_min_ was an independent positive predictor (P < 0.05) and SOFA score on TDM day as an independent negative predictor (P < 0.05) of clinical efficacy. Stratification by SOFA score revealed that this exposure-response relationship was markedly modified by disease severity. In the subgroup with less severely ill patients (SOFA score<6), the positive association between CAZ concentration and clinical efficacy was markedly strengthened (P < 0.001), with ROC analysis identifying an optimal predictive CAZ threshold of 34.79 mg/L (AUC = 0.829). Regarding safety, the overall incidence of adverse drug reactions (ADRs) was 15.03%, primarily elevated transaminases, acute kidney injury, and hematological abnormalities. A concentration-dependent risk for ADRs was established, with a CAZ concentration of 54.84 mg/L identified as the optimal predictive cut-off.

**Conclusion:**

This retrospective analysis provides evidence that optimizing CAZ-AVI plasma concentrations is crucial for improving outcomes in critically ill patients, and that this relationship is significantly modulated by disease severity, necessitating a severity-adapted therapeutic drug monitoring (TDM) strategy.

## 1 Background

Multidrug-resistant Gram-negative bacilli (MDR-GNB), including pathogens such as *Klebsiella pneumoniae*, *Escherichia coli*, *Acinetobacter baumannii*, represents a formidable and escalating challenge for global public health (Collaborators., 2024). Infections caused by these pathogens are closely associated with high morbidity, mortality, and substantial healthcare burdens, particularly among critically ill patients in intensive care units (ICU) (MacVane, 2017; Macesic et al., 2025). For infections due to MDR-GNB, conventional antibiotics are often ineffective, resulting in severaly limited treatment options.

Ceftazidime-avibactam (CAZ-AVI), a novel β-lactam/β-lactamase inhibitor combination, has emerged as a critical therapeutic option for infections caused by MDR-GNB, including complicated intra-abdominal infections, urinary tract infections, and hospital-acquired pneumonia (Shirley, 2018). It exerts a potent inhibitory effect via its avibactam component on Ambler class A, class C and some class D β-lactamases, thereby restoring the antibacterial activity of ceftazidime against a variety of drug-resistant bacteria (Ehmann et al., 2012). Based on animal models and Phase II/III clinical trials, the pharmacokinetic/pharmacodynamic (PK/PD) targets established for CAZ-AVI are as follows: for ceftazidime, the target is 50% fT > MIC (the percentage of the dosing interval that free drug concentration remains above the minimum inhibitory concentration); for avibactam, the target is 50% fT > CT (the percentage of the dosing interval that free drug concentration remains above the threshold concentration required to inhibit β-lactamases), CT = 1–2.5 mg/L (Carmeli et al., 2016; Shirley, 2018; Torres et al., 2018).

However, the PK parameters of antibacterial agents in critically ill patients are highly variable and unpredictable, which may limit the direct applicability of established PK/PD targets. This variability stems from significant alterations in drug distribution and clearance caused by severe illness, pathophysiological changes, and the use of organ support therapies such as continuous renal replacement therapy (CRRT) and extracorporeal membrane oxygenation (ECMO). Furthermore, the patient’s intrinsic disease severity, often quantified by tools like the Sequential Organ Failure Assessment (SOFA) score, not only directly influences prognosis but may also modulate drug exposure (Veiga and Paiva, 2018; Abdul-Aziz et al., 2020). To date, the relationship between disease severity and CAZ-AVI plasma concentrations has not been firmly established. Therefore, this study aims to investigate the correlation of the plasma concentrations of CAZ-AVI and disease severity, as well as clinical efficacy, and to provide evidence for the future development of therapeutic drug monitoring (TDM) strategy for CAZ-AVI in critically ill patients.

## 2 Methods

### 2.1 Study design

In a retrospective, single-center cohort study, we included patients admitted to the ICU of Nanjing Drum Tower Hospital between October 2021 and November 2025 who received CAZ-AVI and underwent therapeutic drug monitoring.

### 2.2 Patient eligibility

Eligible patients were identified through the hospital’s electronic medical record system. Inclusion criteria were (1): age ≥18 years, (2), treatment with CAZ-AVI for ≥48 h in an intensive care setting, (3), availability of trough concentration monitoring after at least 48 h of therapy (4). When a patient had multiple valid concentration measurements, the mean value was used for analysis. Exclusion criteria included (1): incomplete clinical or laboratory data, (2), pregnancy, (3), known allergy to CAZ-AVI, (4), blood sampling for CAZ-AVI concentration performed within 24 h after CRRT initiation, (5), blood sampling performed within 48 h after a dose adjustment.

### 2.3 Assessment of clinical and microbiological efficacy

Both clinical and microbiological efficacy were assessed according to the Chinese Technical Guidelines for Clinical Trials of Antimicrobial Drugs and were evaluated on day 7 after discontinuation of CAZ-AVI therapy. An effective outcome was defined as the complete resolution or return to normal of all baseline infection-related symptoms and signs. An ineffective outcome was defined as the persistence, incomplete resolution, or worsening of baseline symptoms/signs; the development of new infection-related symptoms/signs; and/or the initiation of rescue or alternative antimicrobial therapy against the original infection. Microbiological eradication was defined as the absence of the original pathogen from the original infection site in post-treatment cultures. Presumed eradication was assigned to clinically cured patients for whom follow-up cultures could not be obtained or collection was deemed excessively invasive. Non-eradication was defined as persistent isolation of the original pathogen at the original infection site after treatment. Presumed non-eradication was assigned to clinically ineffective patients in whom follow-up culture was not performed or feasible. For the purpose of analysis, eradication and presumed eradication were combined to calculate the overall eradication rate, while non-eradication and presumed non-eradication were combined to calculate the overall non-eradication rate.

Safety assessment including liver and kidney function and hematological parameters was based on laboratory tests performed every 2–3 days during treatment, with the first occurrence of an abnormal value after treatment initiation recorded as the event onset. To distinguish drug-induced toxicity from sepsis-related organ dysfunction, we applied the Naranjo adverse drug reaction probability scale to each patient with laboratory abnormalities; only events classified as “possible” or above (Naranjo score ≥1) were defined as adverse drug reactions (ADRs). In addition, we performed a lag-time analysis to compare the timing between the occurrence of the high steady-state CAZ concentration of ≥54.82 mg/L and the onset of ADRs, thereby supporting the temporal plausibility of drug causality.

### 2.4 Data collection

Clinical data, including demographics, medical history, medication, infection-related information, infection laboratory indicators, microbiological data, biochemical parameters, SOFA scores (at baseline, during TDM and after treatment), Acute Physiology and Chronic Health Evaluation II (APACHE II) scores were, and organ support status (including CRRT and mechanical ventilation) were collected from the electronic medical records system of Nanjing Drum Tower Hospital.

#### 2.4.1 Dosage regimen

Ceftazidime–avibactam was administered as 2.5 g powder for injection (containing ceftazidime 2 g and avibactam 0.5 g) sourced from GlaxoSmithKline Manufacturing S. p.A. (Italy) or Qilu Pharmaceutical Co., Ltd (China). The daily dosage was adjusted based on renal function, ranging from 0.94 to 7.5 g/d. Specifically, all dosing regimens were determined by the attending physicians according to the drug label and clinical guidelines, using the estimated creatinine clearance (eCrCL). For patients with eCrCL > 50 mL/min, a fixed dose of 2.5 g every 8 h was administered. For patients with eCrCL ≤ 50 mL/min, dose adjustments were made according to the degree of renal impairment: eCrCL 31–50 mL/min: 1.25 g every 8 h; eCrCL 16–30 mL/min: 0.94 g every 12 h; eCrCL 6–15 mL/min: 0.94 g every 24 h. For patients receiving CRRT, the regimen was 1.25 g every 8 h.

#### 2.4.2 Blood sampling and concentration determination

Trough plasma concentrations (C_min_) of ceftazidime and avibactam were measured in blood samples (2 mL) drawn before the next scheduled dose following ≥48 h of therapy. Samples were collected into EDTA tubes, centrifuged (3,000 g, 10 min), and the separated plasma was stored at −20 °C until analysis. Quantification was performed using a validated UPLC-MS/MS system (Waters UPLC I-Class/Xevo TQS). Chromatographic separation used an ACQUITY UPLC BEH C_18_ column (2.1 × 100 mm, 1.7 µm) with a mobile phase of 0.01% formic acid in water (A) and acetonitrile (B) at 0.3 mL/min. Detection was performed using electrospray ionization in positive and negative ion switching mode. The following multiple reaction monitoring (MRM) transitions were applied: for ceftazidime, precursor ion m/z 547.1 → product ion m/z 167.1 (positive mode); for avibactam, precursor ion m/z 264.1 → product ion m/z 95.9 (negative mode). The calibration ranges were 40.0–100 000 mg/L for ceftazidime and 10.0–25 000 mg/L for avibactam. Intra- and inter-batch precision and accuracy were within 15%.

### 2.5 Statistical analysis

Statistical analyses were performed using R (v4.5.1) and RStudio. Continuous variables are presented as mean ± SD or median (IQR), and categorical variables as frequencies (%), based on their distribution (assessed by the Shapiro–Wilk test). Intergroup comparisons utilized t-tests or Mann–Whitney U tests for continuous variables, and χ^2^ or Fisher’s exact tests for categorical variables. To identify independent predictors, variable selection was first performed using least absolute shrinkage and selection operator (LASSO) regression with 10-fold cross-validation (λ.min criterion). Variables retained were then included in a multivariable logistic regression model, with multicollinearity assessed by variance inflation factors (VIF < 5). Given its independent association with efficacy, patients were stratified into two groups based on the median SOFA score on the day of TDM (≥6 vs. <6). Univariate logistic regression was subsequently used within each stratum to assess whether organ dysfunction severity modified the relationship between drug trough concentrations and clinical efficacy. Receiver operating characteristic (ROC) curves were used to evaluate the predictive value of ceftazidime and avibactam concentrations for clinical efficacy in the SOFA <6 subgroup, and of ceftazidime concentration for ADRs in the overall cohort. Optimal cut-offs were determined by the Youden index. Finally, Kaplan-Meier analysis was employed to compare the cumulative incidence of ADRs between patients stratified by the ROC-derived CAZ C_min_ cut-off.

## 3 Results

### 3.1 Demographic data

During the study period, 168 critically ill patients with ceftazidime-avibactam treatment were included, and 11 patients were excluded in this study according to the inclusion and exclusion criteria. 168 patients were stratified into clinical efficacy (n = 108) and non-efficacy (n = 60) groups (Table 1). The groups were well-balanced at baseline, except for the neutrophil percentage, which was significantly higher in one group (P < 0.05). No statistically significant differences were found in age, sex, body weight, APACHE II score, daily dose, treatment duration, infection sites, or microbiology.

**TABLE 1:** Demographic profiles of the patients stratified by treatment efficacy.

Variable	Effective group (N = 108)	Ineffective group (N = 60)	p-value
Demographic			
Age [years]	70.00 (55.00, 79.00)	68.00 (55.25, 76.75)	0.871
Male (%)	79 (73.1)	37 (61.7)	0.171
Body weight [kg]	70.00 (57.50, 70.00)	67.50 (55.00, 70.00)	0.515
ICU stay time [days]	32.50 (21.75, 63.75)	30.50 (21.75, 61.25)	0.863
SOFA score (Day 0)	7.00 (5.00, 10.00)	6.50 (4.75, 10.00)	0.901
APACHE II score (Day 0)	19.00 (15.00, 24.00)	19.50 (15.75, 24.25)	0.487
Lactate (Day 0) [mmol/L]	1.40 (0.98, 2.10)	1.30 (0.98, 2.10)	0.91
CRRT	38 (35.2)	26 (43.3)	0.381
Mechanical ventilation (%)	79 (73.1)	45 (75.0)	0.937
Treatment			
CAZ concentration [mg/L]	39.68 (27.21, 59.36)	26.11 (16.43, 35.90)	**<0.001**
AVI concentration [mg/L]	8.29 (4.47, 14.11)	4.92 (1.77, 10.29)	**0.003**
Daily dose [g]	4.38 (3.75, 7.50)	3.75 (3.75, 7.50)	0.793
Treatment duration [days]	14.00 (11.00, 20.00)	14.00 (9.00, 24.25)	0.88
Concomitant antibiotics (%)	22 (20.4)	9 (15.0)	0.167
Concomitant nephrotoxic medications^a^	48 (44.4)	31 (51.7)	0.461
Concomitant hepatotoxic medications^b^	30 (27.8)	26 (43.3)	0.060
Biochemical parameters			
ALT (Day 0) [U/L]	24.80 (14.67, 39.38)	22.20 (11.62, 43.70)	0.8
AST (Day 0) [U/L]	29.80 (20.05, 44.65)	33.70 (21.55, 43.38)	0.589
Total bilirubin (Day 0) [μmol/L]	14.00 (10.47, 28.50)	16.65 (11.40, 36.38)	0.218
ALB (Day 0) [g/L]	31.85 (28.08, 35.30)	30.50 (28.50, 34.92)	0.537
Urea (Day 0) [mmol/L]	11.60 (7.38, 19.60)	12.20 (6.85, 19.20)	0.935
SCR (Day 0) [μmol/L]	87.00 (59.00, 171.25)	79.50 (52.25, 151.25)	0.53
eGFR^c^ (Day 0) [ml/min/1.73m^2^]	78.40 (37.17, 115.15)	79.15 (35.45, 120.67)	0.798
Infection markers			
CRP (Day 0) [mg/L]	80.30 (47.70, 129.00)	84.25 (46.47, 119.15)	0.945
WBC (Day 0) [×10^9^/L]	9.55 (7.10, 13.87)	10.85 (6.75, 15.72)	0.445
NEUT% (Day 0)	83.80 (76.55, 88.47)	87.50 (79.57, 92.00)	**0.023**
PCT (Day 0) [ng/mL]	0.69 (0.27, 2.25)	0.40 (0.16, 1.85)	0.119
Infection site			
Pulmonary infection (%)	80 (74.1)	43 (71.7)	0.876
Intra-abdominal infection (%)	19 (17.6)	15 (25.0)	0.345
Urinary tract infection (%)	14 (13.0)	9 (15.0)	0.894
Intracranial infection (%)	8 (7.4)	7 (11.7)	0.519
Other infection sites (%)	10 (9.3)	3 (5.0)	0.491
Microbiology			
*Klebsiella pneumoniae* (%)	64 (59.3)	35 (58.3)	1.000
*Pseudomonas aeruginosa* (%)	18 (16.7)	11 (18.3)	0.833
Other pathogenic bacteria (%)	7 (6.5)	4 (6.7)	1.000
Polymicrobial infection (%)	19 (17.6)	10 (16.7)	1.000

Abbreviations: ICU, intensive care unit; SOFA, sequential organ failure assessment; APACHE II, Acute Physiology and Chronic Health Evaluation II; CRRT, continuous renal replacement therapy; CAZ, ceftazidime; AVI, and avibactam; ALT, alanine aminotransferase; AST, aspartate aminotransferase; ALB, albumin; SCR, serum creatinine; eGFR, estimated glomerular filtration rate; CRP, C-reactive protein; WBC, white blood cell count; NEUT%, neutrophil percentage; PCT, procalcitonin.

^a^ Received at least one nephrotoxic medication, including colistin, aminoglycosides, vancomycin, or amphotericin B.

^b^ Received at least one hepatotoxic medication, including azole antifungals, fluoroquinolones, or tetracyclines.

^c^ The eGFR, was calculated using the following formula; eGFR = 142 × min (S_cr_/k, 1)^α^ × max (S_cr_/k, 1)−1200 × 0.9938^Age^ × 1.012 [if female]; where Scr is standardized serum creatinine in mg/dL; κ = 0.7 (female) or 0.9 (male); α = −0.241 (female) or −0.302 (male). The result is expressed in mL/min/1.73 m2.

Bold values indicate statistical significance (p < 0.05).

### 3.2 Correlation between CAZ-AVI plasma concentrations and clinical efficacy

#### 3.2.1 Predictors of clinical efficacy

In this study, patients were classified into clinical efficacy (n = 108) and non-efficacy (n = 60) groups. Significantly higher plasma concentrations of CAZ and AVI were observed in the efficacy group compared to the non-efficacy group (CAZ: 39.68 mg/L [IQR 27.21–59.36] vs. 26.11 mg/L [16.43–35.90], P < 0.001; AVI: 8.29 mg/L [4.47–14.11] vs. 4.92 mg/L [1.77–10.29], P = 0.003).

**FIGURE 1:**
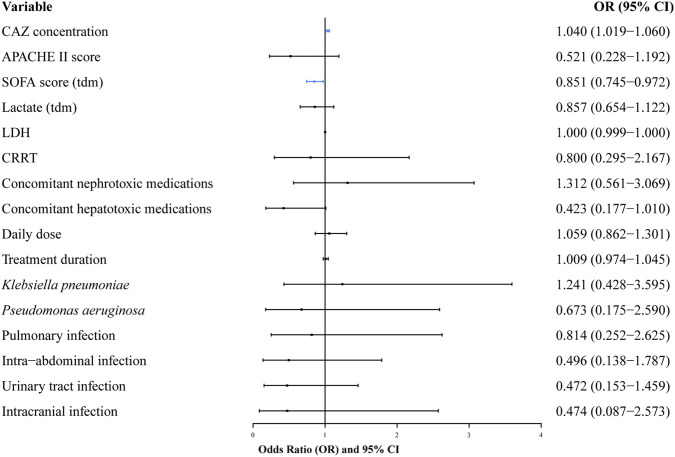
Forest plot showing adjusted OR and 95% CI for factors linked to clinical efficacy. Vertical line = null hypothesis (OR = 1). Blue: statistically significant associations; black: non-significant associations.

Variables screened by LASSO regression, including CAZ concentration, SOFA score on TDM day, APACHE II score, lactate on TDM day, LDH and CKD, together with potential confounding factors including CRRT support, pathogen classification, treatment duration, daily drug dose, concomitant nephrotoxic agents, hepatotoxic medications and infection sites, were incorporated into the multivariate analysis. The results showed that plasma CAZ concentration was an independent positive predictor of clinical efficacy (OR = 1.040, 95%CI: 1.019–1.060, P < 0.001), while SOFA score on TDM day served as an independent negative predictor (OR = 0.851, 95%CI: 0.745–0.972, P = 0.017) (Figure 1). APACHE II score, lactate on TDM day, LDH, CRRT support, pathogen type, treatment duration, daily dosage, concomitant nephrotoxic or hepatotoxic medications, and infection site were not statistically significant. Other pathogen infections, other infection sites and CKD were excluded from the forest plot due to excessively wide 95% CIs, indicating unstable effect estimates.

#### 3.2.2 Pathogen-specific clinical outcomes

In patients infected with metallo-β-lactamase (MBL)-producing isolates, those receiving the combination of CAZ-AVI and aztreonam (ATM) demonstrated a numerically higher clinical cure rate (73.08% vs. 62.68%) and a lower 28-day mortality (15.38% vs. 22.54%) compared to patients infected with non-MBL pathogens treated with CAZ-AVI monotherapy. However, these differences did not reach statistical significance (P = 0.4266 and P = 0.5775, respectively). This trend is mechanistically reasonable, as ATM retains stability against MBLs while avibactam protects it from hydrolysis by coexisting serine β-lactamases, thereby enabling synergistic activity. The lack of statistical significance may be attributable to the limited sample size or confounding factors, warranting further investigation in larger cohorts.

For different MDR-GNB pathogens, comparative analysis between *carbapenem-resistant K. pneumoniae (CRKP)* and *carbapenem-resistant Pseudomonas aeruginosa (CRPA)* groups showed no significant differences in clinical efficacy (64.6% vs. 62.1%, P = 0.9726) or mortality (20.2% vs. 24.1%, P = 0.8429). Notably, bacterial clearance rate was significantly lower in *CRPA* group (41.4% vs. 66.7%, P = 0.0252).

### 3.3 Correlation between CAZ-AVI plasma concentrations and disease severity

**FIGURE 2:**
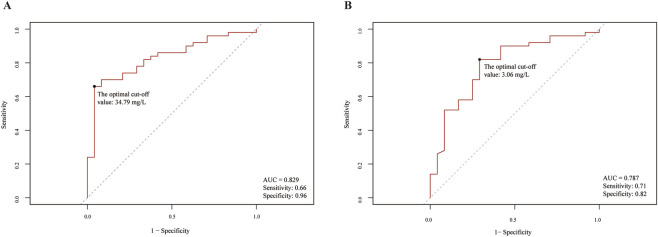
Receiver operating characteristic (ROC) curve of plasma ceftazidime (CAZ) and avibactam (AVI) concentration for predicting clinical efficacy in the subgroup with SOFA score < 6 on the day of therapeutic drug monitoring (TDM). **(A)** Ceftazidime; **(B)** Avibactam.

To investigate whether the severity of organ dysfunction modulates the exposure-efficacy relationship, we stratified patients based on their SOFA score (≥6 vs. <6), given its independent association with clinical outcome in the multivariate model. In the univariate logistic regression analysis of the entire cohort, a higher plasma trough concentration of CAZ (OR 1.0234 per mg/L, 95% CI 1.0084–1.0403, P = 0.003), but not avibactam AVI (OR 1.0396, 95% CI 0.9910–1.0955, P = 0.126), was significantly associated with improved clinical efficacy. However, stratified analysis revealed a critical modification by disease severity. In the subgroup with lower organ dysfunction (SOFA score<6), the positive association between CAZ concentration and clinical efficacy was strengthened (OR 1.0818, 95% CI 1.0361–1.1298, P < 0.001). Moreover, a significant association between AVI trough concentration and efficacy emerged in this subgroup (OR 1.3005, 95% CI 1.0941–1.5457, P = 0.003). Conversely, in the subgroup of patients with higher organ dysfunction (SOFA score≥6), no significant association was found for either CAZ or AVI concentration (Table 2). We subsequently performed ROC analysis to assess the predictive value of the CAZ concentration specifically within the SOFA <6 subgroup (Figure 2). It demonstrated a good predictive performance, with an AUC of 0.829. The optimal plasma CAZ concentration cut-off value was determined to be 34.79 mg/L, which corresponded to a sensitivity of 0.66 and a specificity of 0.96.

**TABLE 2:** Association between plasma ceftazidime (CAZ) and avibactam (AVI) concentrations and clinical efficacy, stratified by SOFA score on the day of TDM.

Grouping	N	CAZ		AVI	
		OR (95% CI) P-value		OR (95% CI) P-value	
Overall	168	1.0234 (1.0084–1.0403)	0.003	1.0396 (0.9910–1.0955)	0.126
Group 1 (SOFA≥6)	94	1.0155 (0.9953–1.0360)	0.134	1.0145 (0.9546–1.0785)	0.645
Group 2 (SOFA<6)	74	1.0818 (1.0361–1.1298)	<0.001	1.3005 (1.0941–1.5457)	0.003

To address whether infection site influenced the identified effective concentration threshold, we performed a subgroup analysis in patients with lower severity of illness (SOFA < 6) using the threshold of 34.79 mg/L. The results showed that a significant association between achieving the concentration target and clinical efficacy was observed only in the pulmonary infection subgroup (n = 40): 80.0% vs. 35.0%, p = 0.009. For intra-abdominal (n = 7), urinary tract (n = 4), intracranial (n = 7), and other infection sites (n = 3), sample sizes were too small to allow reliable statistical comparison, and no significant associations were detected.

### 3.4 Safety evaluation

The overall incidence of ADRs was 13.7% (23/168), primarily elevated transaminases (n = 14), acute kidney injury (n = 6), hematological abnormalities (n = 3). ROC curve analysis was performed to evaluate the predictive value of CAZ C_min_ for ADR occurrence. A trough concentration of 54.82 mg/L was identified as the optimal predictive cut-off value (AUC = 0.790).

To distinguish drug-induced toxicity from sepsis-related organ dysfunction, we performed a lag-time analysis. Among 15 patients who reached the CAZ threshold (≥54.82 mg/L) and experienced an ADR, high concentration preceded ADR in 53.3% (8/15), occurred simultaneously in 33.3% (5/15), and followed ADR in 13.3% (2/15). The median SOFA score in the reverse-sequence group was 3.5 (IQR 3.0–4.0) and in the simultaneous group was 4.0 (IQR 3.0–6.5), neither of which was higher than that in the “high concentration first” group (median 7.5, IQR 5.3–11.0), further suggesting that drug exposure preceded organ injury rather than being secondary to worsening sepsis.

**FIGURE 3:**
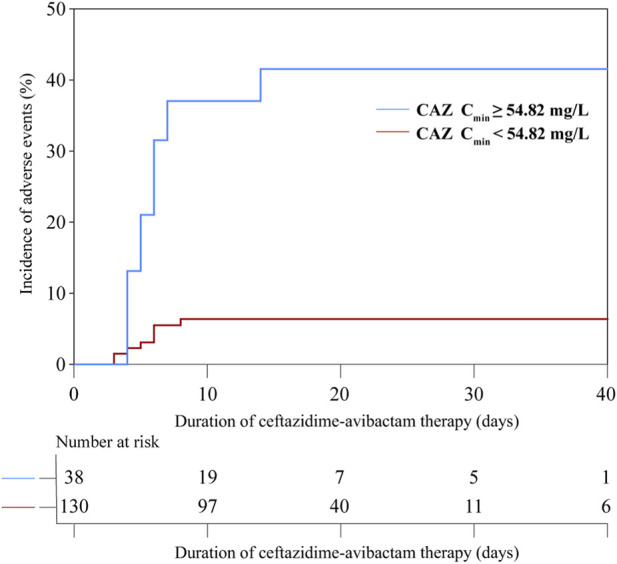
Kaplan-Meier curve of ceftazidime-avibactam treatment duration and incidence of adverse events.

Kaplan-Meier survival analysis further revealed a significant concentration-dependent risk for ADR development (P < 0.05). Patients with a CAZ C_min_ above the defined threshold of 54.82 mg/L had a markedly higher cumulative incidence of ADRs compared to those with concentrations below the threshold. The incidence curve in the high-concentration group reached a plateau at approximately 10 days (Figure 3). The median time to ADR onset from treatment initiation was 5 days.

## 4 Discussion

This study demonstrated that serum concentrations of CAZ-AVI were associated with clinical efficacy in critically ill patients. Specifically, In the overall cohort, a significant correlation was observed between the clinical efficacy and C_min_ of ceftazidime, but not of avibactam; however, a significant association emerged in the subgroup with SOFA <6. That is, in patients with low SOFA (<6), renal function is relatively intact, and drug clearance primarily depends on glomerular filtration; thus, AVI exposure levels better reflect the balance between actual dosing and elimination, making the association with efficacy more apparent. In contrast, among patients with high SOFA, multiple organ failure leads to extreme pharmacokinetic derangements (e.g., increased volume of distribution, reduced clearance, altered protein binding), often accompanied by concurrent use of vasoactive agents and CRRT. These factors likely obscure the direct relationship between AVI concentration and treatment response. More importantly, our analysis showed that the severity of illness at the time of TDM, as quantified by the SOFA score, was a key influencing factor. In the “mild-to-moderate disease” group (SOFA<6), drug exposure emerged as a predominant, modifiable determinant of successful treatment. ROC analysis revealed that this therapeutic target value holds significant predictive value for patients with less severe disease. The clinical effective threshold concentration of CAZ was 34.79 mg/L, with an AUC of 0.829, suggesting good predictive performance. This finding provides a clear quantitative target for precision dosing adjustment in this specific patient population.

As a time-dependent β-lactam drug, the effect of ceftazidime is related to the percentage of time that free drug concentration remains above the minimum inhibitory concentration (MIC) of the targeted pathogen (% fT > MIC). On the other hand, the PK/PD index of avibactam was defined as the free time above a critical concentration (CT) below which sufficient inhibition of ceftazidime was lost (% fT > CT) (Ehmann et al., 2012; Zhanel et al., 2013; Gatti et al., 2024b). For critically ill patients, the PK/PD target is ceftazidime 100% fT > 4–8 × MIC and avibactam 100% fT > CT = 4 mg/L. In this trial, all causative pathogens were susceptible to CAZ-AVI, with an inhibition zone diameter of ≥21 mm (corresponding to a minimum inhibitory concentration [MIC] of≤8/4 mg/L) (Roberts et al., 2014; Abdul-Aziz et al., 2020; Gatti et al., 2024a). This study established a target CAZ concentration of 34.79 mg/L for efficacy in patients with mild-to-moderate critical illness (SOFA score<6) treated with CAZ-AVI. This target approximates 4 × MIC and aligns with previously reported PK/PD goals for CAZ-AVI in critically ill populations.

Critically ill patients exhibit profound PK variability and that achieving PK/PD targets is suboptimal in a large proportion of cases, correlating with worse outcomes. Many studies have also identified that PK of β-lactam antibiotics in critically ill patients is highly prone to alterations (De Waele et al., 2014; Abdulla et al., 2020). Increased capillary permeability, organ dysfunction and other factors may exert a significant impact on PK (Veiga and Paiva, 2018; Abdul-Aziz et al., 2020; Duceppe et al., 2021). Our findings suggest that the correlation of the plasma concentrations of CAZ-AVI and clinical efficacy was observed in the less severely ill patient subgroup (lower SOFA). In patients with lower SOFA scores, limited organ dysfunction means that mortality is more directly tied to infection control. Thus, antibiotic exposure becomes the principal adjustable factor for treatment success, making the exposure-response relationship evident. Conversely, in patients with higher SOFA scores, outcomes are dictated by a multitude of complex factors independent of antibiotic action, including irreversible organ injury, diminished immune response, and the efficacy of non-pharmacological interventions (Moreno et al., 2025; Ranzani et al., 2025). Subgroup analysis in lower severity of illness (SOFA < 6) showed that the association between achieving the concentration target and clinical efficacy was present only in pulmonary infections; other infection sites were too few for reliable analysis. For patients with pulmonary infection and lower severity of illness (SOFA<6), our data strongly support the use of TDM to achieve the identified target concentration, as this is a key lever to optimize their prognosis.

It has been reported that higher plasma concentrations of CAZ and AVI was associated with the adverse events, consistent with our study (Huang et al., 2025). This study demonstrated a robust concentration-dependent relationship between plasma ceftazidime trough concentration and the risk of ADR development, indicating that supratherapeutic exposure contributes to toxicity, especially in patients with impaired clearance or on prolonged regimens. The most frequent adverse events, including elevated transaminases, acute kidney injury, and hematologic abnormalities, align with the cephalosporin class profile but reflect a heightened susceptibility under high-exposure conditions, thereby providing a mechanism-informed explanation for their manifestation (Torres et al., 2023; Zhang et al., 2024; Cheng et al., 2020). Given that ADRs typically emerged at a median of 5 days and incidence plateaued around day 10, early and repeated TDM is essential for critically ill patients. This approach is particularly essential in the ICU setting, given the dynamic changes in renal function and organ perfusion that can precipitate unexpected drug accumulation (Lanini et al., 2024; Li et al., 2026). Importantly, our findings highlight the dual role of TDM in CAZ-AVI therapy, establishing it as a strategy to both maximize efficacy and minimize toxicity.

The optimal dosing strategy for ceftazidime-avibactam in patients undergoing continuous CRRT remains poorly defined due to limited pharmacokinetic/pharmacodynamic data. The Sanford Guide recommends a reduced dose of 1.25 g every 8 h for this population (Gilbert et al., 2023). In contrast, a Chinese expert consensus suggests that dosing in patients receiving 24-h CRRT may be aligned with that in patients with normal renal function, typically 2.5 g every 8 h (Soukup et al., 2019). In the present study, we compared these two dosing strategies (1.25 g q8h vs. 2.5 g q8h) in CRRT patients (Blood flow: 150–200 mL/min, 150 mL/min mostly; replacement fluid rate: 2000 mL/h). No significant differences were observed between the two groups in terms of clinical cure rate, bacterial clearance, or 28-day mortality. However, the incidence of ADRs was significantly higher in the high-dose group (36.4% vs. 7.3, P = 0.029). This finding is likely attributable to increased drug exposure, as a greater proportion of patients in the 2.5 g q8h group achieved plasma ceftazidime concentrations exceeding the previously established ADR threshold of 54.82 mg/L, which was associated with a significantly higher cumulative incidence of toxicity. Collectively, these results suggest that a reduced dose of 1.25 g q8h may offer a safer alternative for patients undergoing CRRT, achieving a reasonable balance between efficacy and safety.

This study also has some limitations. First, the data were collected retrospectively in a single center. Second, the study relied on serum concentrations of CAZ-AVI, which may not accurately reflect its concentrations at the site of infection in different organs and tissues. Third, given the limited sample size, the study is subject to a complex and diverse set of influencing factors.

## 5 Conclusion

In conclusion, this retrospective study suggests that optimizing plasma concentrations of CAZ-AVI is critical for clinical success in critically ill patients. Notably, we demonstrate that the exposure-response relationship is markedly influenced by the severity of illness. This finding necessitates a refined, severity-stratified therapeutic drug monitoring strategy to personalize dosing and maximize therapeutic benefit while minimize risk.

## Data Availability

The original contributions presented in the study are included in the article/supplementary material, further inquiries can be directed to the corresponding author.
